# Antioxidant Activity and Anti-Apoptotic Effect of the Small Molecule Procyanidin B1 in Early Mouse Embryonic Development Produced by Somatic Cell Nuclear Transfer

**DOI:** 10.3390/molecules26206150

**Published:** 2021-10-12

**Authors:** Wei Gao, Tingting Yu, Guomeng Li, Wei Shu, Yongxun Jin, Mingjun Zhang, Xianfeng Yu

**Affiliations:** 1 Jilin Provincial Key Laboratory of Animal Model, College of Animal Science, Jilin University, Changchun 130062, China; wellkao@163.com (W.G.); jyx0429@126.com (Y.J.); mjzhang@jlu.edu.cn (M.Z.); 2 Group of Non-Human Primates of Reproductive and Stem Cell, Kunming Institute of Zoology, CAS, Kunming 650203, China; yutingting@mail.kiz.ac.cn (T.Y.); liguomeng@mail.kiz.ac.cn (G.L.); wishsjh@126.com (W.S.)

**Keywords:** procyanidin B1, mouse, SCNT, embryo, reactive oxygen species

## Abstract

As an antioxidant, procyanidin B1(PB1) can improve the development of somatic cell nuclear transfer (SCNT) embryos; PB1 reduces the level of oxidative stress (OS) during the in vitro development of SCNT embryos by decreasing the level of reactive oxygen species (ROS) and increasing the level of glutathione (GSH) and mitochondrial membrane potential (MMP). Metabolite hydrogen peroxide (H_2_O_2_) produces OS. Catalase (CAT) can degrade hydrogen peroxide so that it produces less toxic water (H_2_O) and oxygen (O_2_) in order to reduce the harm caused by H_2_O_2_. Therefore, we tested the CAT level in the in vitro development of SCNT embryos; it was found that PB1 can increase the expression of CAT, indicating that PB1 can offset the harm caused by oxidative stress by increasing the level of CAT. Moreover, if H_2_O_2_ accumulates excessively, it produces radical-(HO-) through Fe^2+/3+^ and damage to DNA. The damage caused to the DNA is mainly repaired by the protein encoded by the DNA damage repair gene. Therefore, we tested the expression of the DNA damage repair gene, OGG1. It was found that PB1 can increase the expression of OGG1 and increase the expression of protein. Through the above test, we proved that PB1 can improve the repairability of DNA damage. DNA damage can lead to cell apoptosis; therefore, we also tested the level of apoptosis of blastocysts, and we found that PB1 reduced the level of apoptosis. In summary, our results show that PB1 reduces the accumulation of H_2_O_2_ by decreasing the level of OS during the in vitro development of SCNT embryos and improves the repairability of DNA damage to reduce cell apoptosis. Our results have important significance for the improvement of the development of SCNT embryos in vitro and provide important reference significance for diseases that can be treated using SCNT technology.

## 1. Introduction

Somatic cell nuclear transfer (SCNT) is a technology that transfers the nuclei of non-pluripotent differentiated somatic cells into mature oocytes, so that the transplanted oocytes have pluripotency and can develop into normal individuals. SCNT technology can be used to obtain embryonic stem cells derived from nuclear transfer (ntESCs); ntESCs were first obtained from mice [1], and then, scientists obtained ntESCs from rhesus monkeys [2]. Then, scientists used their experience in rhesus monkeys to obtain fibroblasts using fetuses or infants [3]. Both normal humans and type 1 diabetes (T1D) patients have produced ntESCs (nuclei of ntESC cell lines as donors and nuclei of adult somatic cells as donors for nuclear transfer) [4,5]. Studies have shown the potential use of ntESCs in cell replacement therapy [5]. Reproductive cloning has great potential to expand the population of important agricultural economic animals and save endangered animals without sacrificing donor animals [6,7,8,9]. Another potential application of SCNT technology is the generation of new animal models for human diseases [10,11]. Because of the huge application potential of nuclear transfer, in addition to improving the efficiency of nuclear transfer from the perspective of epigenetic modification, it is also very important to explore other aspects to improve the efficiency of nuclear transfer.

Oxidative stress (OS) is a challenging problem in embryo culture and development in vitro. When the embryos are exposed outside the body for manipulation, such as during in vitro fertilization (IVF) [12], intracytoplasmic sperm injection (ICSI) [13], parthenogenetic activation (PA) [14], and SCNT [15], they are subjected to oxidative stress. Under these conditions, embryos produce higher levels of reactive oxygen species (ROS) and reduce the levels of GSH [16], with disruption of the mitochondrial membrane potential [17]. Due to the increase in oxidative stress, a large number of free radicals (such as the hydroxyl radical (-OH)) and non-radical species (such as hydrogen peroxide (H_2_O_2_)) are produced in cells [18,19]. Again, free radical and non-radical species cause oxidative stress, among which H_2_O_2_ is the most typical non-radical species. H_2_O_2_ can be converted to -OH by Fe^2+^/Cu^+^ [18]. Free radicals can cause DNA damage and induce cell apoptosis. In order to enhance the antioxidant capacity of somatic cell nuclear transfer embryos, improve the in vitro development ability of somatic cell nuclear transfer embryos, and reduce the apoptosis level of somatic cell nuclear transfer embryos, it is very necessary to select antioxidants with strong antioxidant activity to be added to the in vitro culture system of somatic cell nuclear transfer embryos.

Procyanidin B1(PB1) is a small-molecule compound, extracted from a variety of plants [20,21,22,23], especially grapes [24,25,26], and it has strong antioxidant effects [22,24,27,28,29]. The antioxidant function of PB1 works by reducing the LPS-induced production of ROS and inhibiting extracellular signal-regulated kinase (ERK)1/2 and IkB kinase beta (IKKb) activity [30]. Additionally, the antioxidant function of PB1 also operates via the indirect activation of the nuclear factor E2-related factor 2 (Nrf2) antioxidant response element (ARE) signaling pathway, which reduces the in vitro and in vivo oxidant levels in HepG2 cells [22]. The antioxidant effects of PB1 can also reduce neuronal death via the attenuation of the activation of caspase-3 by inhibiting the activation of caspase-8 and caspase-9 [28]. Additionally, procyanidin B1 also has anti-inflammatory [30,31], and anti-tumor [32] effects. Furthermore, the antioxidant procyanidin B1 contained in grape extracts protects from chronic metabolic diseases such as diabetes and hypertension, and also prevents the development of chronic kidney disease (CKD) and cardiovascular disease [33]. Thus, PB1 has beneficial effects on health and disease due to its antioxidant effects, and PB1 also benefits the in vitro development of PA embryos [29]. So, PB1 may be a potential and beneficial small molecules drug in relation to SCNT embryo development.

## 2. Results

### 2.1. Determination of the Concentration of PB1 and the Effect of 50 μM PB1 on the Development of SCNT Embryos

**Figure 1:**
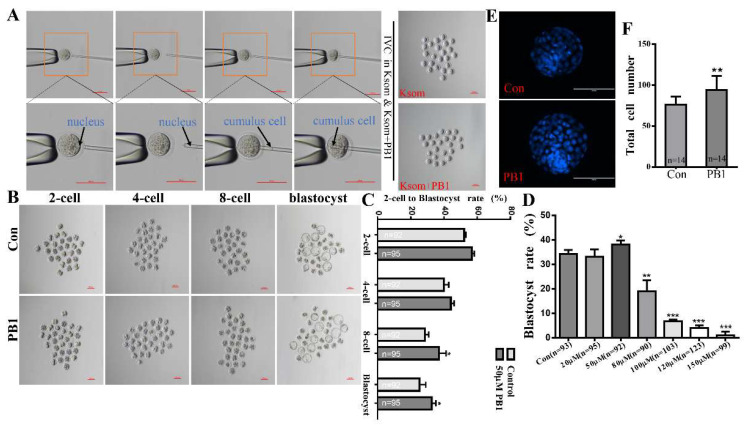
Effects of PB1 on SCNT embryo development. (**A**) Images of SCNT experimental protocol. Scale bar = 100 μm. Below is the display picture of the magnification. Scale bar = 200 μm. (**B**) Picture of 2-cell to blastocyst rates for control and 50 µM PB1 groups. (**C**) Histogram of 2-cell, 4-cell, 8-cell, and blastocyst rate for control and 50 µM PB1 groups. (**D**) Shows the blastocyst rate of control and 20, 50, 80, 100, 120, or 150 µM PB1-exposed groups. (**E**) Picture of blastocyst total cell numbers for control and 50 µM PB1 groups. (**F**) Histogram of blastocyst total cell numbers for control and 50 µM PB1 groups. Values shown are mean ± standard deviation of three independent experiments. * *p* < 0.05, ** *p* < 0.01 and *** *p* < 0.001. *p* < 0.05 indicates a significant difference between the two groups.

SCNT embryos (Figure 1A) were cultured in KSOM media supplemented with 0, 20, 50, 80, 100, 120, and 150 µM PB1 to determine the blastocyst rate. Supplemented 50 µM PB1 significantly increased the blastocyst rate compared with the control group (38.12% ± 1.55% vs. 34.26% ± 1.60%, Figure 1D).

Supplemented 50 µM PB1 affected the development of SCNT embryos; at the eight-cell and blastocyst stage, the eight-cell and blastocyst rate were significantly increased compared with the control group (36.90% ± 4.36% vs. 27.34% ± 2.04%, *p* < 0.05; and 32.65% ± 2.46% vs. 25.27% ± 3.78%, *p* < 0.05, Figure 1B,C).

In regard to the total blastocyst cell numbers in SCNT embryos of cultured with KSOM medium supplemented with 0 and 50 µM PB1, the result showed that the group with supplemented 50 µM PB1 total blastocyst cell numbers were significantly increased compared with the control group (93.86 ± 17.52 vs. 76.00 ± 10.18, *p* < 0.01, Figure 1E,F).

#### 2.1.1. ROS, GSH and JC-1 Ratio Levels in Two-Cell, Four-Cell, Eight-Cell and Blastocyst Embryos Cultured in KSOM Medium Supplemented in 50 µM PB1

**Figure 2:**
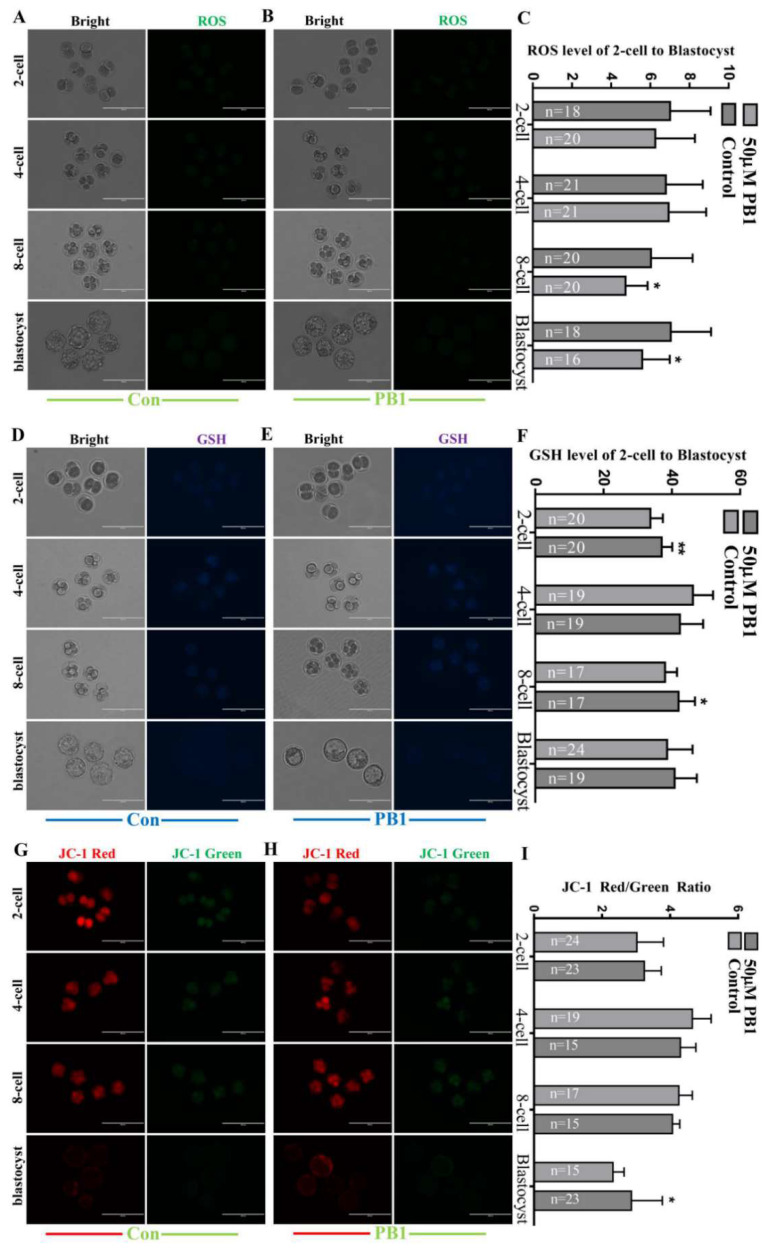
ROS, GSH and MMP levels in 2-cell, 4-cell, 8-cell, and blastocyst embryos. (**A**) Intracellular H2DCFDA-stained (ROS) in 2-cell, 4-cell, 8-cell, and blastocyst embryos of control group. (Scale bar = 200 μm). (**B**) Intracellular H2DCFDA-stained (ROS) in 2-cell, 4-cell, 8-cell, and blastocyst embryos of 50 µM PB1 group. (Scale bar = 200 μm). (**C**) The ROS signal intensity of control group and 50 µM PB1 group. (**D**) Intracellular CMF2HC-stained (GSH) in 2-cell, 4-cell, 8-cell, and blastocyst embryos of control group. (**E**) Intracellular CMF2HC-stained (GSH) in 2-cell, 4-cell, 8-cell, and blastocyst embryos of 50 µM PB1 group. (Scale bar = 200 μm). (**F**) The GSH signal intensity of control group and 50 µM PB1 group. (**G**) JC-red and JC-green in 2-cell, 4-cell, 8-cell, and blastocyst embryos of control group. (Scale bar = 200 μm). (**H**) JC-red and JC-green in 2-cell, 4-cell, 8-cell, and blastocyst embryos of 50 µM PB1 group. (Scale bar = 200 μm). (**I**) The JC-1 ratio (JC-red signal intensity to JC-green signal intensity) of control group and 50 µM PB1 group. The experiment was repeated three times, and values shown are mean ± standard deviation. * *p* < 0.05, ** *p* < 0.01.

At the two-cell stage, the GSH levels were significantly higher than those in the control group (37.03 ± 3.10 vs. 33.70 ± 3.65, *p* < 0.01, Figure 2D–F). At the four-cell stage, there were no significant results. At the eight-cell stage, the GSH levels significantly increased compared with the control group (41.99 ± 4.80 vs. 38.03 ± 3.52 pixels per embryo, *p* < 0.05, Figure 2D–F) and the ROS levels significantly decreased compared with the control group (4.74 ± 1.12 vs. 6.04 ± 2.12 pixels per embryo, *p* < 0.05, Figure 2A–C). JC-1 red represents the JC-1 polymer in the mitochondrial membrane, JC-1 green represents the JC-1 monomer outside the mitochondrial membrane, and JC-1 ratio is the ratio of JC-1 red to JC-1 green, representing the level of mitochondrial membrane potential (MMP). The decrease in MMP level is a landmark event in the early stage of apoptosis. At the blastocyst stage, the JC-1 ratio levels significantly increased compared with the control group (2.86 ± 0.91 vs. 2.32 ± 0.33 pixels per embryo, *p* < 0.05, Figure 2G–I) and the ROS levels significantly decreased compared with the control group (5.59 ± 1.40 vs. 7.25 ± 2.05 pixels per embryo, *p* < 0.05, Figure 2A–C).

#### 2.1.2. The Levels of Catalase (CAT) in Two-Cell, Four-Cell, Eight-Cell and Blastocyst Embryos and the DNA Damage Repairability of PB1

**Figure 3:**
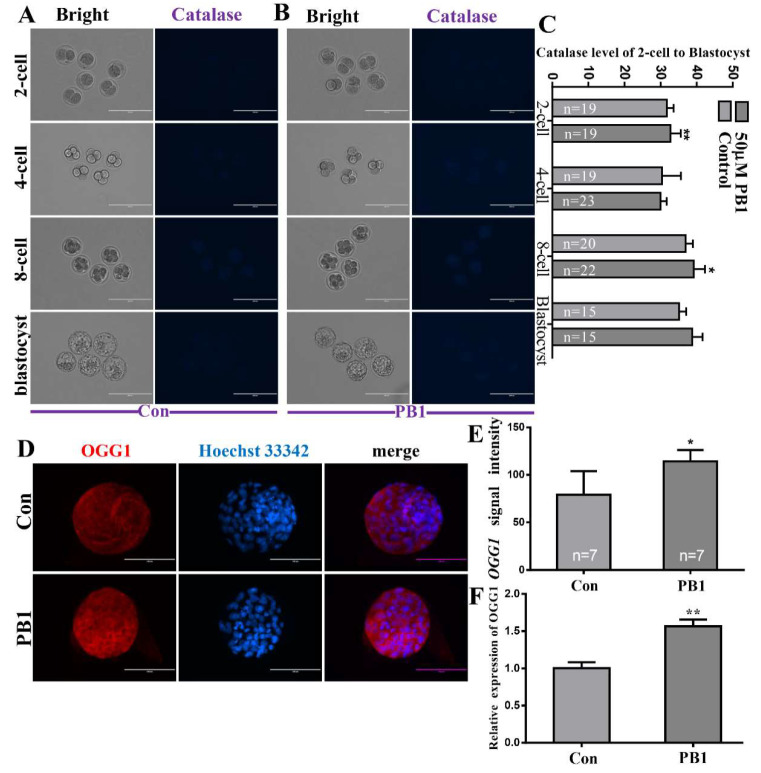
The levels of CAT in 2-cell, 4-cell, 8-cell, and blastocyst embryos and the DNA damage repairability of PB1. (**A**) The CAT levels in 2-cell, 4-cell, 8-cell, and blastocyst embryos of control group. (Scale bar = 200 μm). (**B**) The CAT levels in 2-cell, 4-cell, 8-cell, and blastocyst embryos of 50 µM PB1 group. (Scale bar = 200 μm). (**C**) The CAT signal intensity of control group and 50 µM PB1 group. (**D**) OGG1 levels in blastocyst, with proteins labeled with red fluorescence and blue indicating nuclei. (Scale bar = 100 μm) (**E**) Signal strength of OGG1 protein expression. (**F**) Relative expression levels of OGG1 mRNA in blastocyst. The experiment was repeated three times, and values shown are mean ± standard deviation. * *p* < 0.05, ** *p* < 0.01.

At the two-cell and eight-cell stage, the CAT levels of the group cultured in KSOM medium supplemented in 50 µM PB1 were significantly higher than the control group (39.20 ± 3.07 vs. 36.92 ± 2.06 pixels per embryo, *p* < 0.01; 38.71 ± 2.94 vs. 35.13 ± 1.96 pixels per embryo, *p* < 0.01, Figure 3A–C).

To determine the DNA damage repairability of PB1, we detected the OGG1 mRNA and protein expression in blastocysts; the results showed that the OGG1 mRNA expression was significantly increased and protein expression was increased in the 50 µM PB1 group compared with the control group (114.27 ± 11.86 vs. 79.12 ± 24.82 pixels per embryo, *p* < 0.05, Figure 3D,E, respectively).

#### 2.1.3. Apoptosis of Blastocysts Cultured in KSOM Medium Supplemented in 50 µM PB1

**Figure 4:**
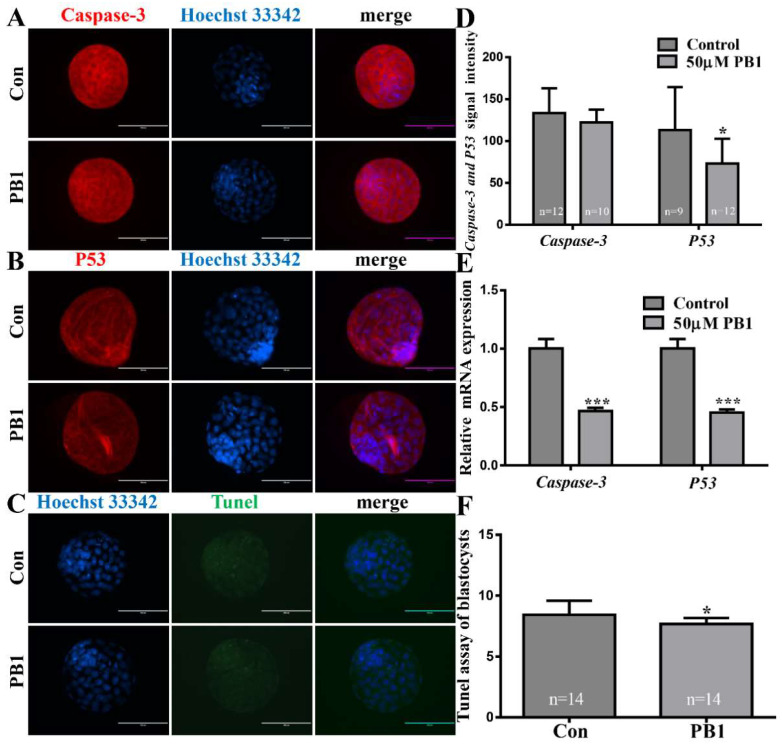
Apoptosis levels of blastocyst. (**A**) Caspase-3 and P53 levels in blastocyst, with proteins labeled with red fluorescence and blue indicating nuclei. Scale bar = 200 μm. (**B**) P53 levels in blastocyst, with proteins labeled with red fluorescence and blue indicating nuclei. Scale bar = 200 μm. (**C**) dUTPs labeled with green fluorescence and blue, indicating nuclei, in blastocysts. Scale bar = 100 μm. (**D**) Signal strength of caspase-3 and P53 protein. (**E**) Relative caspase-3 and P53 mRNA expression levels in blastocysts. (**F**) Percentage of apoptotic in blastocysts. Values indicate mean ± standard deviation of three independent experiments. * *p* < 0.05, *** *p* < 0.001.

To determine their ability to inhibit apoptosis of PB1, we evaluated P53 and caspase-3 mRNA and protein expression through immunofluorescence staining. There were no significant differences in the caspase-3 protein expression. However, the caspase-3 mRNA expression significantly decreased. Additionally, the P53 mRNA expression also significantly decreased. The expression of P53 protein in blastocysts decreased in the 50 µM PB1 group compared with the control group (73.47 ± 29.36 vs. 113.33 ± 50.85 pixels per embryo, *p* < 0.05, Figure 4A,B,D, respectively). The TUNEL results show that the 50 µM PB1 group showed a significant decrease in apoptosis level compared with the control group (7.67 ± 0.50 vs. 8.43 ± 1.15 pixels per blastocyst, *p* < 0.05, Figure 4C,F, respectively).

## 3. Discussion

Based on the results of previous studies [22,25,29], we can determine that PB1 is a small-molecule drug with antioxidant effects. In addition, we can confirm that it can improve the developmental ability of embryos during early development [29]. The in vitro development of embryos is inefficient compared to embryos derived in vivo [34]; embryos cultured in vitro exhibit increased ROS levels, especially SCNT embryos during the process of micromanipulation in vitro [35]. SCNT technology in embryos can also cause damage to mitochondrial membrane potential, DNA, and gene and protein expression [35,36]. This experiment focuses on the effect of PB1 on the early development of SCNT embryos to reduce the level of OS in SCNT embryos development. Firstly, the optimal concentration of PB1 during SCNT embryos development was selected. In view of the poor treatment effect of 100 μM (the concentration used in the in vitro maturation of pig embryos) [29] and the lower rate of blastocysts, we believe that the processing time for SCNT embryos in mice is longer; it takes 3.5 days. We finally determined the best concentration to be 50 μM. In the SCNT embryos treated at this concentration, the eight-cell rate and the blastocyst rate were significantly improved, and the number of blastocyst cells was more than that of the control group without PB1. The above results can show that PB1 at a concentration of 50 μM promotes the development of mouse embryos in vitro.

Scientists engaged in embryo development research have been committed to finding ways to reduce the oxidative stress of embryo development in vitro. The most common method is the addition of antioxidants to in vitro embryo culture medium, and the most representative one is vitamin C (VC); VC can reduce excessive ROS levels and reduce DNA damage and apoptosis [37]. With the progress of chemical extraction methods and the continuous discovery of new chemical molecules, increasing numbers of antioxidants have been discovered and used to improve oxidative stress in biological research, especially in the field of embryonic development, such as asiatic acid [38] and melatonin [39]. Using PB1 as an antioxidant, we analyzed the effect of PB1 on the level of ROS in SCNT embryos and found that in the late stage of embryonic development (eight-cell and blastocyst stage), the level of ROS showed a significant decrease, indicating that PB1 can play a role in scavenging ROS during development, and that it takes time to accumulate so that it can play a role. The finding that PB1 can reduce the level of active oxygen is consistent with the results of previous studies [29,30].

As an important regulatory metabolite in cells, GSH has an antioxidant effect which plays an important reducing role and is significantly increased during the two-cell and eight-cell stage of SCNT embryo development during PB1 processing, which is consistent with the previous results found in the maturation of pig oocytes [29], and the results are consistent with those found in intestinal normal-like cells [40]. The improvement of GSH level occurred earlier, in the two-cell stage. From this, we guessed that PB1 first increased the GSH level, and GSH then played a role to reduce the ROS level in the eight-cell and blastocyst stages. In addition, the endogenous source of ROS is mainly mitochondria. MMP plays a key role in mitochondrial respiration. When MMP is depolarized, a large amount of ROS is produced [29]. Additionally, GSH can also attenuate the depolarization of MMP [41]. Our results show that when the MMP in the blastocyst increases, there is a corresponding decrease in ROS in the blastocysts. In addition, in the process of redox in the cell, GSH and GSSG (L-Glutathione Oxidized) are continuously circulated under the action of the glutathione cycle. H_2_O_2_ can be degraded into H_2_O through the process of converting GSH to GSSG, thereby reducing oxidative stress. Therefore, we believe that this process may be due to PB1 increasing the conversion rate of GSSG to GSH, and the underlying mechanism may be that PB1 promotes the activity of glutathione-related invertase, and decreases glutathione reductase level.

In the process of oxidative stress, some metabolic by-products, such as H_2_O_2_, can be produced [42]. These metabolic by-products of oxidative stress can cause serious damage to cells. H_2_O_2_ can cause cell DNA damage and cell apoptosis [43]. The decrease in CAT levels causes an increase in H_2_O_2_ levels [44]. We analyzed the level of CAT that can decompose H_2_O_2_, and after PB1 treatment, the level of CAT in SCNT embryos (two-cell and eight-cell stage) showed a significant increase. The above results can show that PB1 increases the expression level of CAT, which in turn can reduce the production of H_2_O_2_. The possible mechanism is that PB1 activates the expression of the CAT gene, and the activated CAT gene promotes the level of CAT. CAT can reduce the H_2_O_2_ level, so the H_2_O_2_ harm is reduced, which reduces DNA damage and cell apoptosis, thereby reducing DNA damage and cell apoptosis. The blastocyst stage is the final stage of early embryo development and the longest PB1 treatment stage. Therefore, we tested the blastocyst DNA damage repairability and the level of blastocyst apoptosis in the blastocyst stage. Since the protein encoded by OGG1 (DNA damage repair gene) can protect DNA from ROS damage [45], we detected the expression of the OGG1 gene in blastocysts. We found that the expression of the OGG1 gene in blastocysts treated with PB1 was significantly increased compared with the group without PB1, and protein expression was also significantly increased compared with the group without PB1. We also carried out a correlation analysis on the level of apoptosis of blastocyst stage cells. Although immunofluorescence staining showed that the protein level of caspase-3 did not decrease significantly, the level of mRNA showed a significant decrease in the expression of caspase-3. Another representative apoptotic protein, P53, showed a significant reduction in protein and mRNA levels. TUNEL analysis also showed a significant reduction in apoptosis. The above results can prove that PB1 can enhance the embryo’s DNA repairability and reduce cell apoptosis.

In summary, our results show that PB1 can reduce the level of oxidative stress during the early development of SCNT embryos, improve the embryo’s ability to remove oxidative stress metabolites, and enhance the embryo’s DNA damage repairability, thereby reducing the level of apoptosis and promoting the development ability of SCNT embryos.

## 4. Materials and Methods

### 4.1. Ethics Statement

The mice used for our experiments were kept in a comfortable environment (12 h of light, 12 h of darkness, 20 °C, moderate humidity). Our experiments complied with the Animal Care and Use Committee of Jilin University, Changchun, China (Grant No. SY202009068).

### 4.2. Reagents and Animals

We purchased all chemicals and reagents from Sigma-Aldrich (St. Louis, MO, USA), unless stated otherwise.

We used 8-week-old female offspring (B6D2F1) produced after mating of C57 BL/6J (female) and DBA/2 (male) as experimental animals for SCNT.

### 4.3. Drug Treatment and Experimental Design

Based on our and others’ previous research on porcine [30] and on mouse embryos, we analyzed the concentration of PB1 by treating the embryos with 0, 20, 50, 80, 100, 120, and 150 μM. We found 50 μM to be the most suitable concentration. We dissolved PB1 in KSOM solution to make a storage solution with a concentration of 500 mM and stored it at −20 °C. When we came to use the working solution, we first diluted it to 500 μM with KSOM solution, and then diluted it to the required concentration.

Regarding MII oocytes after SCNT, we chose good SCNT embryos and transferred them to the new in vitro culture (IVC) KSOM medium (NaCl 555 mg/100 mL, KCl 18.5 mg/100 mL, KH_2_PO_4_ 4.75 mg/100 mL, MgSO_4_ 7H_2_O 4.95 mg/100 mL, CaCl_2_ 2H_2_O 25 mg/100 mL, NaHCO_3_ 210 mg/100 mL, Glucose 3.6 mg/100 mL, Na-Pyruvate 2.2 mg/100 mL, DL-Lactic Acid, sodium salt 0.174 mL/100 mL, 10 mM EDTA 100 μL/100 mL, Streptomycin 5 Penicillin 6.3 mg/100 mL, 0.5% phenol red 0.1 mL/100 mL, L-Glutamine 14.6 mg/100 mL, MEM Essential Amino Acids 1 mL/100 mL, MEM Non-essential AA 0.5 mL/100 mL, BSA 100 mg/100 mL) and KSOM medium supplemented with 50 μM of PB1. During the IVC process, we counted the 2-cell, 4-cell, 8-cell, and blastocyst embryos at 24, 48, 48–60, 72–84 h after SCNT.

### 4.4. SCNT Protocol and Embryonic IVC

Each female B6D2F1 mouse was injected with 7.5 IU PMSG (pregnant mare serum gonadotropin; Merck Millipore, San Francisco, CA, USA) at 6:30 p.m. Forty-eight hours later, the mice were injected with 7.5 IU hCG (human chorionic gonadotropin; Sigma, St. Louis, MO, USA). After 13–15 h, we killed the mice by cervical dislocation, and the oviducts were removed and put into HCZB [CZB stock medium 94 mL (500 mL of CZB stock medium: special media ultra-pure water (470 mL), NaCl (2380 mg), KCl (180 mg), MgSO_4_·7H_2_O (145 mg), EDTA·2Na (20 mg), D-Glucose (500 mg), KH_2_PO_4_ (80 mg), Na-Lactate (2.65 mL)), PVA (10 mg), Hepes (476 mg), NaHCO_3_ (42 mg), CaCl_2_·2H_2_O 100× stock (42 mg), CaCl_2_·2H_2_O 100× stock (1 mL), Na-Pyruvate (3.7 mL), Glutamax (1mL)]. Next, the cumulus–oocyte complexes (COCs) were picked from the magnum tubae uterinae of the oviducts; we detached cumulus cells from B6D2F1 COCs with 2% hyaluronidase melted in HCZB, and washed them 3 times using HCZB. B6D2F1 MII oocytes were prepared for SCNT. The restoration, activation, and culture of embryos were carried out at 37 °C with 5% CO_2_/95% air conditions, and all media overlaid with mineral oil were prepared for use in an incubator.

SCNT protocol: We removed the B6D2F1 MII oocytes nucleus using a micromanipulator in HCZB containing 0.5 μg/mL CB [HCZB + CB(Cytochalasin B)], and we used cumulus cells of B6D2F1 mice as donor cells. After that, we injected cumulus cells into B6D2F1 MII oocytes without nuclei. All manipulations were completed within 18 h after the HCG injection. Next, we put the SCNT embryos in KSOM medium to recover for 1 h, and then we put the SCNT embryos into Ca^2+^-free CZB containing 10 μM of Sr^2+^ and 0.5 μg/mL of CB for 6 h. After that, we transferred SCNT embryos to a KSOM medium containing 0.5 nM of TSA(trichostatin A) for 4 h. Finally, we transferred SCNT embryos into fresh KSOM medium. The KSOM culture medium supplemented with 50 μM of PB1 was used as the experimental group, and the KSOM culture medium without any addition was used as the control group.

### 4.5. Assay of Total Blastocyst Cell Numbers

SCNT blastocysts were collected in order to count the total cell numbers. All blastocysts were washed three times using PBS-PVA (phosphate-buffered saline mixed with 1 g/L of PVA) and fixed with 4% (*w*/*v*) paraformaldehyde; fixed blastocysts were washed three times again and incubated in 5 μg/mL of Hoechst 33342 for 10 min at 37 °C. After that, blastocysts were washed three times again; washed blastocysts were mounted on a glass slide under a glass coverslip, and photographed using EVOS FL.

### 4.6. Immunofluorescence Staining and Quantitative Real-Time Polymerase Chain Reaction (Q-PCR) Protocol

Blastocysts (the control group and 50 µM PB1-treated group) were fixed with 4% (*w*/*v*) paraformaldehyde solution after being washed three times with PBS-PVA, and incubated with 0.2% (*v*/*v*) Triton X-100 for 15 min. Then, after being washed 3 times, all fixed blastocysts were incubated with 1% (*w*/*v*) BSA for 1 h. After they were fixed, blastocysts were incubated with anti-P53 (1:100; Abcam, Cambridge, UK), anti-caspase-3 (1:100; Abcam) and anti-OGG1 (1:100; GeneTex, Irvine, CA USA) antibodies at 4 °C overnight. The next day, they were washed 3 times and incubated with a secondary antibody (1:100; CY3-goat anti-rabbit; Boster Biological Technology, Wuhan, China) at 37 °C for 2 h. After being incubated with a secondary antibody and washed three times, we placed the blastocysts in Hoechst 33342 for 10 min at room temperature. Blastocysts were washed three times again; washed blastocysts were mounted on a glass slide under a glass coverslip, and photographed using EVOS FL (Waltham, MA, USA).

Total mRNA was extracted from about 20–30 blastocysts using a microRNA extraction kit (cat. 74181, Qiagen, Dusseldorf, Germany) and reverse transcribed into cDNA using a reverse transcription kit (Tiangen Biotech, Beijing, China). We added SYBR green fluorescent dye (Tiangen Biotech), cDNA, ddH_2_O, and primers (Table 1) to a 96-well PCR Cell PCR-plate using an RT-PCR instrument (Eppendorf, Hamburg, Germany). The RT-PCR cycles were as follows: pre-denaturation at 95 °C for 15 min, 95 °C for 10 s (denaturation), 60 °C for 20 s (annealing), and 72 °C for 30 s (extension) for 45 cycles. The β-actin gene was used for standardization. Three independent experiments were performed; we used the 2 − ΔΔCt (ΔΔCt = ΔCt (case) − ΔCt (control)) method to calculate relative mRNA expression. The primer sequences used for real-time PCR are shown in Table 1.

### 4.7. TUNEL Method

Blastocysts (control group and 50 µM PB1 treated group) were removed from the KSOM medium, washed with PBS-PVA three times, and fixed with 4% (*w*/*v*) paraformaldehyde solution for 10 min, then washed 3 times, and transferred to 0.2% (*v*/*v*) Triton X-100 for 15 min. Then, we washed the blastocysts 3 times, and we incubated the blastocysts with TdT and fluorescein-conjugated dUTPs (In Situ Cell Death Detection Kit; Roche, Mannheim, Germany) in the dark for 30 min at 37 °C. After that, we washed the embryos 3 times and transferred the blastocysts to Hoechst 33342 for 5 min at 37 °C, and they were washed again three times with PBS-PVA. The processed blastocysts were fixed on a glass slide, and observed and photographed using EVOS FL.

### 4.8. ROS, GSH and MMP Level Assay

Two-cell, four-cell, eight-cell, and blastocyst embryos were collected to measure ROS, GSH and MMP levels. All the embryos (2-cell, 4-cell, 8-cell, and blastocyst) were washed 3 times to examine ROS levels, embryos were incubated with PBS-PVA containing 10 µM 2,7-dichlorodihydrofluorescein diacetate (H2DCFDA; Solarbio Life Sciences, Beijing, China) for 5 min, and after being washed three times, they were photographed using EVOS FL (green fluorescence, UV filters, 490 nm, Waltham, MA, USA). To detect GSH levels, 2-cell, 4-cell, 8-cell, and blastocyst embryos were incubated with PBS-PVA containing 10 µM 4-chloromethyl-6,8-difluoro-7-hydroxycoumarin (CMF2HC, Solarbio Life Sciences, Beijing, China) for 5 min, then washed three times, and photographed using EVOS FL (blue fluorescence, UV filter, 370 nm, Waltham, MA, USA). To detect MMP levels, we incubated all cells with PBS-PVA containing JC-1 fluorescent probe (Solarbio Life Sciences, Beijing, China) at 37 °C for 30 min. Cells were photographed using a fluorescence microscope (EVOS FL, Waltham, MA, USA) with 490 nm (green fluorescence) and 530 nm (red fluorescence) excitation, and the MMP was calculated as the ratio of red fluorescence (corresponding to activated mitochondria) to green fluorescence (corresponding to less activated mitochondria, J-monomer).

### 4.9. CAT Levels Method

Two-cell, four-cell, eight-cell and blastocyst embryos were collected to measure CAT levels; all embryos were washed 3 times, then incubated with 200 μL of solution 1 mixed with 1 μL of solution 2 (cat. BC0205, Solarbio Life Sciences, Beijing, China) for 5 min; after that, we placed the embryos in KSOM medium to recover for 5 min, and photographed them using fluorescence microscope (EVOS FL, Waltham, MA, USA) (blue fluorescence, UV filter) excitation.

### 4.10. Statistical Analysis

We repeated each experiment at least three times and analyzed the data using SPSS 20.0 software (IBM, Armonk, NY, USA). We used the Student’s t-test to analyze comparisons of the two groups (treatment and control groups) and the ANOVA test to analyze comparisons of more than two groups and Dunnett’s test to analyze Figure 1D. *p* < 0.05 was considered statistically significant.

## 5. Conclusions

The above research results indicate that as an antioxidant small molecule, PB1 can reduce the oxidative stress level during the early development of SCNT embryos by reducing the level of ROS and increasing the level of GSH. Additionally, it can improve the ability of embryos to scavenge free radicals such as H_2_O_2_, improve DNA damage repairability, and reduce the expression level of embryonic apoptosis markers, as well as reduce the level of apoptosis of SCNT embryonic blastocysts, thereby reducing the level of apoptosis. All the results support the idea that PB1 can be used as a supplement to promotes the development of SCNT embryos.

## Figures and Tables

**Table 1:** Primer sequences used for real-time PCR.

Gene		Primer	Sequence	Annealing
		Forward	CTGCCTAGCAGCATGAGACAT	
*OGG1*	[45]			61 °C
		Reverse	CAGTGTCCATACTTGATCTGCC	
		Forward	CAGCCAAGTCTGTGACTTGCACGTAC	
*P53*	[46]			61 °C
		Reverse	CTATGTCGAAAAGTGTTTCTGTCATC	
		Forward	CCAACCTCAGAGAGACATTC	
*Caspase3*	[47]			61 °C
		Reverse	TTTCGGCTTTCCAGTCAGAC	
		Forward	GGGAAATCGTGCGTGACATT	
*β-actin*	[48]			61 °C
		Reverse	GCGGCAGTGGCCATCTC	

## Data Availability

The data presented in this study are available on request from the corresponding author.

## Sample Availability

Samples are available from the authors.

## References

[1] Rideout W.M., Hochedlinger K., Kyba M., Daley G.Q., Jaenisch R. (2002). Correction of a genetic defect by nuclear transplantation and combined cell and gene therapy. Cell.

[2] Byrne J.A., Pedersen D.A., Clepper L.L., Nelson M., Sanger W.G., Gokhale S., Wolf D.P., Mitalipov S.M. (2007). Producing primate embryonic stem cells by somatic cell nuclear transfer. Nature.

[3] Tachibana M., Amato P., Sparman M., Gutierrez N.M., Tippner-Hedges R., Ma H., Kang E., Fulati A., Lee H.S., Sritanaudomchai H. (2013). Human embryonic stem cells derived by somatic cell nuclear transfer. Cell.

[4] Chung Y.G., Eum J.H., Lee J.E., Shim S.H., Sepilian V., Hong S.W., Lee Y., Treff N.R., Choi Y.H., Kimbrel E.A. (2014). Human somatic cell nuclear transfer using adult cells. Cell Stem Cell.

[5] Yamada M., Johannesson B., Sagi I., Burnett L.C., Kort D.H., Prosser R.W., Paull D., Nestor M.W., Freeby M., Greenberg E. (2014). Human oocytes reprogram adult somatic nuclei of a type 1 diabetic to diploid pluripotent stem cells. Nature.

[6] Beyhan Z., Iager A.E., Cibelli J.B. (2007). Interspecies nuclear transfer: Implications for embryonic stem cell biology. Cell Stem Cell.

[7] Loi P., Ptak G., Barboni B., Fulka J., Cappai P., Clinton M. (2001). Genetic rescue of an endangered mammal by cross-species nuclear transfer using post-mortem somatic cells. Nat. Biotechnol..

[8] Kamimura S., Inoue K., Ogonuki N., Hirose M., Oikawa M., Yo M., Ohara O., Miyoshi H., Ogura A. (2013). Mouse cloning using a drop of peripheral blood. Biol. Reprod..

[9] Wakayama S., Ohta H., Hikichi T., Mizutani E., Iwaki T., Kanagawa O., Wakayama T. (2008). Production of healthy cloned mice from bodies frozen at −20 degrees C for 16 years. Proc. Natl. Acad. Sci. USA.

[10] Doudna J.A., Charpentier E. (2014). Genome editing. The new frontier of genome engineering with CRISPR-Cas9. Science.

[11] Hsu P.D., Lander E.S., Zhang F. (2014). Development and applications of CRISPR-Cas9 for genome engineering. Cell.

[12] Kumar S., Mishra V., Thaker R., Gor M., Perumal S., Joshi P., Sheth H., Shaikh I., Gautam A.K., Verma Y. (2018). Role of environmental factors & oxidative stress with respect to in vitro fertilization outcome. Indian J. Med. Res..

[13] Ashibe S., Miyamoto R., Kato Y., Nagao Y. (2019). Detrimental effects of oxidative stress in bovine oocytes during intracytoplasmic sperm injection (ICSI). Theriogenology.

[14] Luo D., Zhang J.-B., Li S.-P., Liu W., Yao X.-R., Guo H., Jin Z.-L., Jin Y.-X., Yuan B., Jiang H. (2020). Imperatorin Ameliorates the Aging-Associated Porcine Oocyte Meiotic Spindle Defects by Reducing Oxidative Stress and Protecting Mitochondrial Function. Front. Cell. Dev. Biol..

[15] An Q., Peng W., Cheng Y., Lu Z., Zhou C., Zhang Y., Su J. (2019). Melatonin supplementation during in vitro maturation of oocyte enhances subsequent development of bovine cloned embryos. J. Cell. Physiol..

[16] Takahashi M. (2012). Oxidative stress and redox regulation on in vitro development of mammalian embryos. J. Reprod. Dev..

[17] Al-Zubaidi U., Liu J., Cinar O., Robker R.L., Adhikari D., Carroll J. (2019). The spatio-temporal dynamics of mitochondrial membrane potential during oocyte maturation. Mol. Hum. Reprod..

[18] Poprac P., Jomova K., Simunkova M., Kollar V., Rhodes C.J., Valko M. (2017). Targeting Free Radicals in Oxidative Stress-Related Human Diseases. Trends Pharmacol. Sci..

[19] Firuzi O., Miri R., Tavakkoli M., Saso L. (2011). Antioxidant therapy: Current status and future prospects. Curr. Med. Chem..

[20] Matta F.V., Xiong J., Lila M.A., Ward N.I., Felipe-Sotelo M., Esposito D. (2020). Chemical Composition and Bioactive Properties of Commercial and Non-Commercial Purple and White Acai Berries. Foods.

[21] Zhang X., Li X., Su M., Du J., Zhou H., Li X., Ye Z. (2020). A comparative UPLC-Q-TOF/MS-based metabolomics approach for distinguishing peach (Prunus persica (L.) Batsch) fruit cultivars with varying antioxidant activity. Food Res. Int..

[22] Li T., Li Q., Wu W., Li Y., Hou D.-X., Xu H., Zheng B., Zeng S., Shan Y., Lu X. (2019). Lotus seed skin proanthocyanidin extract exhibits potent antioxidant property via activation of the Nrf2-ARE pathway. Acta Biochim. Biophys. Sin..

[23] Gillmeister M., Ballert S., Raschke A., Geistlinger J., Kabrodt K., Baltruschat H., Deising H.B., Schellenberg I. (2019). Polyphenols from Rheum Roots Inhibit Growth of Fungal and Oomycete Phytopathogens and Induce Plant Disease Resistance. Plant Dis..

[24] Fia G., Bucalossi G., Gori C., Borghini F., Zanoni B. (2020). Recovery of Bioactive Compounds from Unripe Red Grapes (cv. Sangiovese) through a Green Extraction. Foods.

[25] Sano A., Yamakoshi J., Tokutake S., Tobe K., Kubota Y., Kikuchi M. (2003). Procyanidin B1 is detected in human serum after intake of proanthocyanidin-rich grape seed extract. Biosci. Biotechnol. Biochem..

[26] Mao J.T., Xue B., Smoake J., Lu Q.Y., Park H., Henning S.M., Burns W., Bernabei A., Elashoff D., Serio K.J. (2016). MicroRNA-19a/b mediates grape seed procyanidin extract-induced anti-neoplastic effects against lung cancer. J. Nutr. Biochem..

[27] Zuriarrain A., Zuriarrain J., Puertas A.I., Duenas M.T., Ostra M., Berregi I. (2015). Polyphenolic profile in cider and antioxidant power. J. Sci. Food Agric..

[28] Kanno H., Kawakami Z., Tabuchi M., Mizoguchi K., Ikarashi Y., Kase Y. (2015). Protective effects of glycycoumarin and procyanidin B1, active components of traditional Japanese medicine yokukansan, on amyloid beta oligomer-induced neuronal death. J. Ethnopharmacol..

[29] Gao W., Jin Y., Hao J., Huang S., Wang D., Quan F., Ren W., Zhang J., Zhang M., Yu X. (2021). Procyanidin B1 promotes in vitro maturation of pig oocytes by reducing oxidative stress. Mol. Reprod. Dev..

[30] Terra X., Palozza P., Fernandez-Larrea J., Ardévol A., Bladé C., Pujadas G., Salvado J., Arola L., Blay M.T. (2011). Procyanidin dimer B1 and trimer C1 impair inflammatory response signalling in human monocytes. Free Radic. Res..

[31] Xing J., Li R., Li N., Zhang J., Li Y., Gong P., Gao D., Liu H., Zhang Y. (2015). Anti-inflammatory effect of procyanidin B1 on LPS-treated THP1 cells via interaction with the TLR4-MD-2 heterodimer and p38 MAPK and NF-kappaB signaling. Mol. Cell. Biochem..

[32] Na W., Ma B., Shi S., Chen Y., Zhang H., Zhan Y., An H. (2020). Procyanidin B1, a novel and specific inhibitor of Kv10.1 channel, suppresses the evolution of hepatoma. Biochem. Pharmacol..

[33] Zhu J., Du C. (2020). Could grape-based food supplements prevent the development of chronic kidney disease?. Crit. Rev. Food Sci. Nutr..

[34] Ma Y., Gu M., Chen L., Shen H., Pan Y., Pang Y., Miao S., Tong R., Huang H., Zhu Y. (2021). Recent advances in critical nodes of embryo engineering technology. Theranostics.

[35] Su J., Wang Y., Xing X., Zhang L., Sun H., Zhang Y. (2015). Melatonin significantly improves the developmental competence of bovine somatic cell nuclear transfer embryos. J. Pineal Res..

[36] Hwang I.S., Bae H.K., Cheong H.T. (2013). Mitochondrial and DNA damage in bovine somatic cell nuclear transfer embryos. J. Vet. Sci..

[37] Zhang X., Zhou C., Cheng W., Tao R., Xu H., Liu H. (2020). Vitamin C protects early mouse embryos against juglone toxicity. Reprod. Toxicol..

[38] Qi J.J., Li X.X., Diao Y.F., Liu P.L., Wang D.L., Bai C.Y., Yuan B., Liang S., Sun B.X. (2020). Asiatic acid supplementation during the in vitro culture period improves early embryonic development of porcine embryos produced by parthenogenetic activation, somatic cell nuclear transfer and in vitro fertilization. Theriogenology.

[39] Qu P., Shen C., Du Y., Qin H., Luo S., Fu S., Dong Y., Guo S., Hu F., Xue Y. (2020). Melatonin Protects Rabbit Somatic Cell Nuclear Transfer (SCNT) Embryos from Electrofusion Damage. Sci. Rep..

[40] Attanzio A., D’Anneo A., Pappalardo F., Bonina F.P., Livrea M.A., Allegra M., Tesoriere L. (2019). Phenolic Composition of Hydrophilic Extract of Manna from Sicilian Fraxinus angustifolia Vahl and its Reducing, Antioxidant and Anti-Inflammatory Activity in Vitro. Antioxidants.

[41] Kelly-Aubert M., Trudel S., Fritsch J., Nguyen-Khoa T., Baudouin-Legros M., Moriceau S., Jeanson L., Djouadi F., Matar C., Conti M. (2011). GSH monoethyl ester rescues mitochondrial defects in cystic fibrosis models. Hum. Mol. Genet..

[42] Del Rio L.A., Lopez-Huertas E. (2016). ROS Generation in Peroxisomes and its Role in Cell Signaling. Plant Cell Physiol..

[43] Mizutani H., Hayashi Y., Hashimoto M., Imai M., Ichimaru Y., Kitamura Y., Ikemura K., Miyazawa D., Ohta K., Ikeda Y. (2019). Oxidative DNA Damage and Apoptosis Induced by Aclarubicin, an Anthracycline: Role of Hydrogen Peroxide and Copper. Anticancer Res..

[44] Cheng C.H., Ma H.L., Deng Y.Q., Feng J., Jie Y.K., Guo Z.X. (2021). Oxidative stress, cell cycle arrest, DNA damage and apoptosis in the mud crab (Scylla paramamosain) induced by cadmium exposure. Chemosphere.

[45] Mori Y., Ogonuki N., Hasegawa A., Kanatsu-Shinohara M., Ogura A., Wang Y., McCarrey J.R., Shinohara T. (2021). OGG1 protects mouse spermatogonial stem cells from reactive oxygen species in culturedagger. Biol. Reprod..

[46] Kunimura N., Kitagawa K., Sako R., Narikiyo K., Tominaga S., Bautista D.S., Xu W., Fujisawa M., Shirakawa T. (2020). Combination of rAd-p53 in situ gene therapy and anti-PD-1 antibody immunotherapy induced anti-tumor activity in mouse syngeneic urogenital cancer models. Sci. Rep..

[47] De Santis R., Liepelt A., Mossanen J.C., Dueck A., Simons N., Mohs A., Trautwein C., Meister G., Marx G., Ostareck-Lederer A. (2016). miR-155 targets Caspase-3 mRNA in activated miR-155 targets Caspase-3 mRNA in activated macrophages. RNA Biol..

[48] Kong D., Yan Y., He X.Y., Yang H., Liang B., Wang J., He Y., Ding Y., Yu H. (2019). Effects of Resveratrol on the Mechanisms of Antioxidants and Estrogen in Alzheimer’s Disease. BioMed Res. Int..

